# Effectiveness of Generative AI–Enabled Teaching for Clinical Reasoning in Health Care Education: Protocol for a Systematic Review and Meta-Analysis

**DOI:** 10.2196/95709

**Published:** 2026-10-15

**Authors:** Suzanne Johnston, Sonyia McFadden, David H Glass, Claire McFeeters, Joanne Marley

**Affiliations:** 1 Faculty of Life and Health Science School of Health Science Ulster University Belfast, Northern Ireland United Kingdom; 2 School of Computing Ulster University Belfast, Northern Ireland United Kingdom

**Keywords:** generative artificial intelligence, GenAI, health care professional, education, clinical reasoning, systematic review

## Abstract

**Background:**

Clinical reasoning skills are key competencies required by health care professionals. Generative AI (GenAI)–enabled teaching may be able to offer personalized learning; however, its effectiveness compared with traditional teaching methods for developing clinical reasoning capabilities remains underexplored.

**Objective:**

This systematic review and meta-analysis will evaluate, synthesize, and compare the evidence on effectiveness in clinical reasoning capabilities between GenAI-enabled teaching and traditional teaching methods in health care education.

**Methods:**

This review protocol will adhere to the PRISMA-P (Preferred Reporting Items for Systematic Reviews and Meta-Analysis Protocols) guidelines. The population will include health care professionals. The intervention will be the use of GenAI-enhanced teaching methods, with traditional teaching methods as the comparator. The primary outcome will be objective clinical reasoning performance, with relevant validity for the construct, population, and intended purpose. A database search will be conducted across Embase, MEDLINE, CINAHL, Scopus, and Cochrane CENTRAL, supplemented by Google Scholar, reference list, and citation searches. Study designs will include randomized controlled trials (RCTs), non-RCTs, quasiexperimental studies, and cohort studies with suitable controls. Title and abstract screening and full-text screening will be completed by 2 independent reviewers, with arbitration as required. IBM SPSS will be used to combine and calculate the overall effect. A random-effects model will be deployed, with the Hartung-Knapp-Sidik-Jonkman method used, as appropriate, to calculate the CI around the estimated pooled effect. Study heterogeneity will be assessed using *I*^2^ and τ^2^, and where studies are judged to be too diverse, results will be presented narratively. Prediction intervals will be used, if appropriate, to show how the true effect may vary between similar studies. Risk of bias will be reviewed by 2 independent reviewers, with arbitration, as required, using the Risk of Bias 2 (ROB 2) tool for RCTs and the Risk of Bias in Non-Randomized Studies of Interventions (ROBINS-I) tool for non-RCTs, quasiexperimental studies, and interventional cohort studies. Sensitivity analysis will be used to explore possible sources of heterogeneity. A subgroup analysis may include the type and functionality of GenAI used and/or health care professional background.

**Results:**

The review was funded in September 2025. A trial search was completed on MEDLINE in December 2025, with final database searches completed in February 2026. A total of 4831 studies were retrieved, with 2083 duplicates removed. A further 2558 studies were removed at title and abstract screening, with 190 studies undergoing full-text screening and 42 studies included in the final review. Expected completion date of the final review is autumn or winter 2026.

**Conclusions:**

Health care education is rapidly adopting GenAI-enhanced teaching methods, despite inconclusive evidence on their effectiveness. The findings from this review will offer essential insight into the optimization of GenAI-enhanced teaching methods for clinical reasoning within current and future curriculum design.

**Trial Registration:**

PROSPERO CRD420261284944; https://www.crd.york.ac.uk/PROSPERO/view/CRD420261284944

**International Registered Report Identifier (IRRID):**

DERR1-10.2196/95709

## Introduction

### Background

Clinical reasoning, which combines both cognitive and metacognitive skills, is a core competency in health care education and is essential for safe and effective clinical practice [1]. Rigorous approaches to the teaching of all aspects of clinical reasoning are essential to ensure that health care professionals are equipped to manage the complexities of modern health care provision [2,3].

The need to ensure robust clinical reasoning in health care professionals has never been more pressing. Complexity within health care is rising dramatically due to a combination of medical, situational, and systemic factors [4-6]. An increasingly aging population, escalating global burden of disease, and poorly integrated health care systems, against a backdrop of chronic staff shortages and rising staff burnout, have created a “wicked” problem in modern health care [7].

In response, health care educational institutions are experiencing a parallel challenge in absorbing rising student numbers, while simultaneously maintaining high-quality educational experiences that meet the demands of unpredictable and complex clinical settings [3]. These educational priorities are further complicated by operational challenges for higher education providers, with recruitment difficulties, an aging academic workforce, and evidence of a 25% decline in senior clinical educators in medicine over the past decade [8,9].

The emergence of AI, particularly generative AI (GenAI), within health care education, has provided a compelling opportunity for innovative and creative development of clinical reasoning skills [10,11]. The accessibility of large language models (LLMs), such as Open AI’s ChatGPT, offers the potential of personalized, multimodal learning at scale, across a diverse spectrum of learners [12-14]. This aligns with increasing acceptability among health care students regarding the use of LLMs in supporting clinical reasoning development [15,16].

### Prior Work

The use of GenAI in developing components of clinical reasoning has shown early promise across a variety of different teaching methods, with existing systematic reviews looking at (1) GenAI-enabled versus traditional teaching in medical education [17], (2) simulated virtual patients in nursing education [11,18,19], and (3) problem- and case-based learning in medical and dental education [20]. It is notable that existing reviews predominantly feature undergraduate students in the fields of medicine and nursing, with relative underrepresentation of allied health profession education [16] and postgraduate learners [14,17]. These factors, alongside the inclusion of broad components of clinical reasoning, limit generalization of the effectiveness of GenAI-enabled teaching compared with traditional teaching methods [17,20]. The effectiveness of GenAI-enabled teaching of specific clinical reasoning components has been demonstrated in several studies [11,21,22]. Improvement has been demonstrated in diagnostic reasoning and treatment planning in a single-arm study using GenAI-enabled virtual patients, with 62% of students rated as “good” during observation assessments [21]. Custom GPT technology has also demonstrated significant improvements in problem-based learning skills compared with traditional teaching methods, as measured by self-directed learning scores and the Cornell Critical Thinking Test [22].

However, the ability of GenAI to support clinical reasoning in complex clinical scenarios is uncertain. GenAI-enabled teaching methods were explored by Çiçek et al [23], who compared ChatGPT-enabled teaching and feedback with traditional teaching and expert feedback across urological clinical scenarios of varying complexity. No significant difference in outcomes was found for simple scenarios, as assessed by key feature questions, but this appeared to reverse in more complex scenarios, where traditional teaching methods and feedback groups showed superior outcomes. Similarly, Brügge et al [24] highlighted the limitations of LLMs in responding to subtle nonverbal and contextual cues in a simulation setting, factors which commonly add to the complexity of authentic clinical scenarios. These findings raise important questions around the positioning of GenAI in complex clinical reasoning development.

The elements of nuance, ambiguity, uncertainty, and contextual awareness are ill-defined but essential factors required for navigating clinical complexity, which may arguably align more comfortably with human expert capabilities [25]. Understanding how and when to use GenAI-enabled or more traditional teaching methods and in which clinical reasoning domains is a crucial consideration for both current and future curriculum development.

Ambiguous definitions of clinical reasoning, in combination with methodological heterogeneity and unclear reporting methods, limit understanding of the components of clinical reasoning best suited to GenAI-enabled teaching. Supporting frameworks have been developed to facilitate structured reporting of applied AI interventions in medical education, such as the FACETS (form of GenAI; AI use case; context; education focus; technology; SAMR [substitution, augmentation, modification, and redefinition; this is a prior framework to describe integration of technology) framework, to improve the cohesive description of AI interventions [12]. Similarly, Gordon et al [1] developed a reporting framework to support clearer understanding and alignment of clinical reasoning components and assessment.

This systematic review aims to evaluate the effectiveness of GenAI-enabled teaching methods compared with traditional teaching methods on clinical reasoning outcomes in health care education. It will use structured reporting frameworks [1,12] to provide an overview of the GenAI applications being used, the domains of clinical reasoning being developed, and how outcomes are being measured, to inform the quality of reporting in future studies.

In determining the optimal relationship between traditional teaching and GenAI-enabled teaching methods, it is important to recognize the ambiguity that already exists around clinical reasoning pedagogy [1,26,27]. The term clinical reasoning, while a foundational competency stipulated by regulatory health bodies [28-30], has varying definitions, with ongoing debate concerning how it should be taught and how outcomes should be assessed [1,26,31].

Capturing a unified definition of clinical reasoning is problematic given different conceptualizations across the health care professions, with broad assessment descriptors, ill-defined parameters, limited theoretical underpinning, and varying levels of construct validity, further compounded by few explicit descriptions of how clinical reasoning should be incorporated across the lifespan of health care education curricula [1,27,32].

In this review, to enable a broad abstraction of clinical reasoning capabilities, we will adopt the following overarching definition: “a skill, process or outcome wherein clinicians observe, collect, and interpret data to diagnose and treat patients” [2]. This definition allows for multiple, parallel constructs to be considered, including but not limited to, information gathering, diagnostic reasoning, critical thinking, problem solving, clinical decision-making, application of theoretical knowledge, and treatment planning [27].

Given the existing lack of pedagogical cohesion, due consideration needs to be given to the added integration of GenAI-enabled teaching methods to ensure that learning outcomes are not misinterpreted or conflated. LLMs are subsets of GenAI that are becoming increasingly used in health care education due to their proficiency in generating rich, context-specific outputs that mimic humanlike interactions [33-35]. The unique capability of LLMs to produce multimodal, personalized content, alongside impressive performance capabilities in medical knowledge examinations, has accelerated the integration of GenAI into health care curricula [36].

While GenAI potentially provides the environment to enhance cognitive skills, empirical evidence on long-term learning outcomes has not been established, and direct translation to the improvement of clinical reasoning skills has not been proven [37,38]. This ongoing uncertainty supports the need for clear evidence on how and when GenAI could be used to support, enhance or even replace traditional teaching methods for the development of clinical reasoning.

The number of studies exploring the use of GenAI-enabled teaching methods in health professions education is increasing, particularly in the fields of undergraduate medical and nursing education [13,39-41]. However, despite this rise in research activity, a prior scoping review on the use of AI in undergraduate medical education highlighted a notable lack of studies assessing the impact of AI-enabled teaching on clinical reasoning and critical thinking [42].

A previous systematic review and meta-analysis [20] explored the use of AI-enabled tools in problem- and case-based learning in medical and dental education. Mixed outcomes were reported regarding clinical reasoning development, and cautious interpretation was suggested due to the potential for immediate posttest scoring to amplify positive results. These review findings reflect the wide range of individual clinical reasoning components commonly subsumed under the broader definition of clinical reasoning, alongside a similar breadth of applied assessment methods. While the included studies explore facets of clinical reasoning, this is insufficient to suggest competency across all domains of clinical reasoning, and more comprehensive and varied assessment strategies are recommended [20].

GenAI is being increasingly adopted in health care education due to its potential in supporting personalized and adaptive learning [33,35]. However, the effectiveness of GenAI-enabled teaching for developing clinical reasoning skills remains uncertain.

The current evidence on GenAI-enabled health care education highlights common study limitations, including short-term follow-up [20,38,43,44], small sample sizes [38,43], heterogeneity of interventions and outcomes [12,19,20,38], and an overreliance on self-reported outcome measures [18,44,45]. These limitations are further magnified by inconsistent reporting of how GenAI interventions are being deployed to support clinical reasoning development, which components of clinical reasoning are being targeted, and how educational effectiveness is being assessed.

Given the foundational nature of clinical reasoning in health care education, it is imperative that educators are informed by transparent evidence on whether GAI-enabled teaching improves clinical reasoning compared with traditional teaching methods and how interventions can be optimally aligned to enhance these essential skills.

### Objectives

Therefore, the aim of this systematic review will be to evaluate, synthesize and compare the effectiveness of GenAI-enabled teaching methods compared with traditional teaching methods on clinical reasoning capabilities in health care education. Secondary objectives will include describing the clinical reasoning components targeted by GenAI-enabled teaching interventions, the methods used to assess these components, and the ways in which GenAI is integrated into interventional design. The inclusion of theoretical frameworks and/or the iteration of explicit learning objectives will also be explored.

## Methods

### Ethical Considerations

No ethics approval is required for this review as it will be conducted using previously published data. The review will adhere to the PRISMA-P (Preferred Reporting Items for Systematic Reviews and Meta-Analysis Protocols) guidelines [46], full details of which can be found in Multimedia Appendix 1.

### Eligibility Criteria

This review will adopt the population, intervention, comparator, and outcome (PICO) framework to support key components of study design. The study population will include undergraduate and postgraduate students enrolled in health care professions education in higher education institutions. Studies with postregistration learners in clinical settings will be included if there is evidence of a clear focus on the use of GenAI-enabled teaching for the purpose of developing clinical reasoning. Studies in either setting using GenAI-enabled teaching for purposes other than the development of clinical reasoning will be excluded. Laboratory-based studies unrelated to clinical reasoning will be excluded.

The following health care professionals will be included: medical, nursing, dental, pharmacy, and all allied health care professionals recognized by the UK Health and Care Professions Council [28]. There will be no restrictions regarding gender or geographic location. Studies involving non–health care professionals or laypeople will be excluded.

Interventions, including novel interventions that demonstrate clear use of GenAI-enabled technology in health care professions education for the development of clinical reasoning will be included. This will be inclusive of, but not limited to, LLMs; GPTs, including ChatGPT, DALL·E (OpenAI), Claude (Anthropic), Gemini (Google LLC), Bing (Microsoft Corp), Copilot (Microsoft Corp), DeepSeek (Hangzhou DeepSeek Artificial Intelligence Co, Ltd), Grok (SpaceXAI), Midjourney (Midjourney, Inc), Perplexity (Perplexity AI); GenAI-enabled simulation technology; GenAI-enabled virtual patients and/or simulation; GenAI-enabled virtual reality and GenAI-enabled augmented reality, virtual agents; and GenAI-enabled tutoring systems. GenAI-enabled teaching methods delivered face-to-face, online, or hybrid formats, either individually or in groups, will be included. Interventions using GenAI will be defined and reported using the FACETS framework [12].

Studies that do not explicitly indicate that GenAI-enabled technology has been used will be excluded. Any uncertainty regarding the AI functionality included within a study will be discussed with a subject expert (DHG). Studies that use GenAI-enabled tools within clinical settings for clinical care (eg, rehabilitation devices, robotic aids, wearable devices, clinical decision support tools, or image analysis tools) where the main purpose is to support clinical care and efficiency or to provide patient education, will be excluded. Studies that do not identify intentional integration of GenAI-enabled tools into teaching and learning design for the development of clinical reasoning will be excluded. For the purposes of this review, intentional integration will be interpreted as deliberate or targeted use of GenAI for the specific educational purpose of enhancing components of clinical reasoning, as opposed to unrestricted or optional access to GenAI for broader, unspecified learning support.

Comparator groups will include traditional teaching methods of clinical reasoning in health care education including didactic teaching methods, lecture-based teaching, seminars, tutorials, individual and group-based teaching sessions, online and hybrid learning approaches, and case-based and problem-based learning.

The primary outcome will be objectively assessed clinical reasoning performance. Studies will be included that describe established clinical reasoning instruments or methods, with relevant validity for the construct, population, and intended purpose. Studies that focus only on outcomes related to specific psychomotor skill development (eg, specific surgical, technical, or practical skills) will be excluded. Studies that demonstrate assessment of both GenAI-enabled teaching methods of psychomotor skills and nonpsychomotor skills will be included if there is evidence of reported outcome measures specifically targeting clinical reasoning.

It will be reported whether studies have identified a specific definition or component of clinical reasoning and which measurement tool has been used to measure clinical reasoning outcomes, guided by the definitional and methodological assessment framework suggested by Gordon et al [1]. GenAI integration will be guided by the FACETS framework [12]. The time points of assessment will also be identified.

The explicit iteration of specific learning outcomes or theoretical frameworks will be explored**.** Intended learning outcomes will be defined as “the desired intended result from the applied teaching activity” [47]. Theoretical frameworks will be defined as “structures or theories that frame the interpretation, analysis, and meaning of results” [48]. Refer to Table 1 for a summary outline of the inclusion and exclusion criteria.

Included study designs will be RCTs, non-RCTs, quasiexperimental studies, and cohort studies with a control arm. Nonrandomized study designs have been included as a limited number of true RCTs are anticipated. We will exclude case studies, single case series, or studies without a control arm.

**Table 1:** Inclusion and exclusion criteria using the population, intervention, comparator, and outcome (PICO) framework.

PICO element	Inclusion criteria	Exclusion criteria
Population	Undergraduate and postgraduate students enrolled in health care professions education Postregistration learners in clinical settings using GenAI-enhanced teaching methods for clinical reasoning All geographic locations All genders	Non–health care professionals or laypeople
Intervention	GenAI-enabled teaching methods for health care professions education delivered face-to-face, online, or through hybrid learning, individually or in groups	No explicit use of GenAI-enhanced teaching methods Unrestricted use of GenAI for general learning support GenAI-enabled tools where the main purpose is to support clinical care, efficiency, or patient education
Comparator	Traditional teaching methods of clinical reasoning	—^a^
Outcome	Quantitative outcomes from studies that describe established clinical reasoning instruments or methods for a defined construct, population, and purpose	Outcomes related to psychomotor skills only Studies that focus only on laboratory-based data

^a^Not applicable.

### Information Sources

The publication search will be limited to studies published from 2022 to February 2026. This limitation has been set to coincide with GenAI accessibility, including the public release of DALL·E and ChatGPT in 2022 [35]. This decision has also been informed by a previous systematic review and meta-analysis [17] on the use of GenAI- based teaching methods, with publication limitations set between 2014 and 2025, but with all studies included for final analysis dating between 2024 and 2025.

Electronic searches will be undertaken with the help of an information specialist in the following databases from 2022 to February 2026: Embase, MEDLINE, CINAHL, Scopus, Cochrane CENTRAL, and Google Scholar. To identify any additional relevant studies, reference lists of papers undergoing full-text review will be manually searched alongside a forward citation search using the Google Scholar citation index. A Google Scholar alert will be implemented to capture additional relevant studies up to the point of data synthesis. The PRISMA-S (Preferred Reporting Items for Systematic Reviews and Meta-Analyses literature search extension) framework [49] will support a transparent search strategy, and a completed checklist can be viewed in Multimedia Appendix 2.

No limitations will be placed on language or geographic location. Translation software will be used at the initial screening stage to extract relevant study details and, if relevant studies meet the eligibility criteria, translation services may be engaged for full-text review.

Eligible studies will include peer-reviewed articles that report primary empirical research (randomized and nonrandomized, as applicable). Editorials, commentaries, conference reports, letters, protocols, and nonempirical opinion pieces will be excluded. Higher-quality studies will be considered for meta-analysis, and lower-quality studies will undergo narrative synthesis. All articles retrieved will be documented in the PRISMA (Preferred Reporting Items for Systematic Reviews and Meta-Analyses) diagram, which will be available in the full review paper.

A trial search will be undertaken in MEDLINE (Multimedia Appendix 3) to refine the search strategy prior to undertaking the final systematic searches (Multimedia Appendix 4). Covidence systematic review software will be used to support deduplication, data screening, and population of the PRISMA diagram. Any studies erroneously removed as duplicates will be returned for title and abstract screening. Title and abstract screening will be conducted by 2 independent reviewers, with conflicts unresolved by discussion, being arbitrated by a third reviewer.

The review team will pilot the eligibility criteria on approximately 6 titles and abstracts. Any refinements made to the eligibility criteria following the pilot will be documented. In cases where full-text articles cannot be retrieved, the lead corresponding author will be contacted by SJ. All articles retrieved or sought for retrieval will be documented in the PRISMA diagram.

Two independent reviewers will screen full-text articles for inclusion or exclusion, with any conflicts unresolved by discussion, being arbitrated by a third reviewer. In cases of incomplete data, the primary author will be contacted by SJ, where contact details have been provided. If this is unsuccessful, the study will be identified as incomplete. Interrater reliability will be assessed using Cohen κ coefficient after 20% of studies have been screened at both the title and abstract and full-text stages. Scores of <0.5 will result in a review of the eligibility criteria. Covidence will be used to calculate κ scores.

Data extraction will be undertaken using a customized data extraction table. Details will include the study details and characteristics. Interventions will be reported using the FACETS and clinical reasoning assessment frameworks [1,12]. Data will be extracted by SJ and will be cross-checked and verified by a second member of the research team to ensure the rigor of the data extraction. Refer to Table 2 for a summary outline of the planned data extraction.

**Table 2:** Data extraction table.

Intervention variable	Description
Study details	Author, publication year, study title, and journal
Study characteristics	Type of study, study design, sample size, setting, and geographic location
FACETS reporting framework [12]	F: Form of GenAI A: AI use case (eg, virtual patient) C: context (eg, professional membership, level of learner, and specific area of learning) E: education focus (eg, curriculum, assessment, and feedback) T: technology (eg, ChatGPT and novel GPT) S: SAMR (substitution, augmentation, modification, and redefinition), a previous framework to describe integration of technology
Clinical reasoning assessment framework [1]	Definition of clinical reasoning, evidence of theoretical model informing intervention, assessment methodology, and content validity of chosen assessment method
Study findings	Primary outcome, results, main findings, and limitations
Time from intervention to assessment	Using identified time points of interest

### Synthesis of Results

If adequate data are available for each outcome, SPSS software (version 31.0; IBM Corp) will be used to combine and calculate the overall effect. The Cochrane Handbook for systematic reviews of interventions will be followed to support use of the correct statistical methods [50]. Dichotomous data will be assessed using the relative risk (95% CI). Continuous data will be reported using the mean difference for studies using similar scales and the standardized mean difference (SMD) for studies in which similar domains are being studied using different measurement scales (95% CI).

A random-effects model will be used to assess between-study variance (τ^2^) [51,52]. If between-study variance exceeds 0, with 3 or more studies eligible for inclusion, the Hartung-Knapp-Sidik-Jonkman method will be used to calculate the CI around the estimated pooled effect. This will support a more precise estimate of predicted variation between studies [51]. If 3 or more studies are eligible for inclusion but there is an imbalance in the sizes of the included studies, a modified Knapp-Hartung method will be adopted [53]. However, if between-study variance is 0, and less than 3 studies are included in the meta-analysis, the Wald method will be adopted [51].

Statistical heterogeneity will be assessed using *I*^2^, τ^2^ and interpreted alongside the direction and magnitude of effects and the clinical and methodological diversity of the included studies [51]. Where studies are judged to be too diverse for a pooled estimate to be meaningful, results will be presented narratively in tables.

If the following conditions are present, (1) >5 studies are eligible for review, (2) a normal distribution can be assumed, and (3) there is no clear evidence of funnel plot asymmetry, prediction intervals may be used to show the true effect variance between similar studies [51,52], where possible, SEs will be changed to SDs allowing data to be included within a meta-analysis.

Where a study includes more than one eligible intervention arm, arms will be combined into a single group where appropriate, or the comparison most relevant to the review question will be selected, to avoid double-counting. Cluster randomized trials will be included where results have been adjusted for clustering or will be adjusted using an intracluster correlation coefficient in line with the Cochrane Handbook [50].

Primary outcomes will include objectively assessed clinical reasoning performance measures, using tools with appropriate content validity. As the proposed inclusion criteria include a range of quantitative metrics, studies that measure similar domains, but different measurement tools will be grouped using SMD. When several instruments are used to measure the same outcome domain, the following decision rule will be applied to ensure the inclusion of the most reliable and comprehensive measures, with known construct validity for clinical reasoning. Initial consideration will be given to studies that use key feature questions [2], extended matching questions [2], script concordance tests [2], or objective structured clinical examinations, and mini-clinical evaluation exercises [2], with parameters iterated for clinical reasoning domains. Measures of critical thinking, or parallel concepts that apply assessor led strategies will then be considered, followed by applied theoretical knowledge using tools with reported content validity measures. Self-reported measures of aligned domains will be considered together. If data are reported in other formats such as medians or ranges, data will be tabulated, and a narrative report provided.

### Risk of Bias Assessment

The Risk of Bias 2 (ROB 2) tool [54] will be used to assess risk of bias in RCTs, and the Risk of Bias in Non-Randomized Studies of Interventions (ROBINS-I) tool [55] will be used for nonrandomized studies, quasiexperimental, and interventional cohort studies. Risk of bias assessment will be conducted by 2 independent reviewers, with conflicts unresolved by discussion, being arbitrated by a third reviewer. The robvis tool will be used to support visualization of the risk of bias [56].

Sensitivity analysis will be undertaken to explore possible sources of heterogeneity, for example, studies with a high risk of bias. Where applicable, a funnel plot will be used to explore publication bias. A subgroup analysis may be performed on the type and functionality of GenAI tools, in recognition of the evolving technical ability of GenAI tool development within the time frame of this proposed study. Health care professional background may also be included in a subgroup analysis.

Quality of evidence will be assessed by 2 independent reviewers using the GRADE (Grading of Recommendations Assessment, Development, and Evaluation) framework [57] and will be judged on risk of bias, consistency, directness, precision, and publication bias. The quality will be rated as high, moderate, low, and very low. In the event of conflict unresolved by consensus, a third reviewer will arbitrate the final decision.

## Results

The review was funded in September 2025 by the Northern Ireland Department of the Economy, as part of a PhD project at Ulster University. A trial search was conducted in a single database (MEDLINE) in December 2025, with the assistance of an information specialist (Multimedia Appendix 3). This search included the names of the AI platforms specified in the proposed PICO. The results of this search yielded a high proportion of studies examining or comparing the reasoning performance of individual platforms, but limited studies regarding the effectiveness of GenAI interventions in developing clinical reasoning skills. As a result, the research team, in collaboration with an information specialist and guided by a previously published search strategy [17], adapted the final search strings (Multimedia Appendix 4).

The final searches were therefore conducted in the Embase, MEDLINE, CINAHL, Scopus, and Cochrane CENTRAL databases and Google Scholar in February 2026, with the application of planned filters, including a date range from 2022 to February 2026 and no limitation by language. The final search identified 4831 studies, with 2083 (43%) duplicate studies removed and 2748 (57%) studies screened at the title and abstract stage by 2 independent reviewers. A total of 2558 studies were excluded at the title and abstract stage, with 190 studies undergoing full-text review and 42 studies included in the final review.

A full PRISMA diagram will be included in the final review, with expected completion in autumn or winter 2026. Dissemination strategies will be through academic publication and conference proceedings.

## Discussion

### Anticipated Findings

Current evidence can only offer limited conclusions about the role that GenAI might play in supporting the development of clinical reasoning competencies [38,42]. There remains persistent ambiguity around clinical reasoning pedagogy, how GenAI is being used to support, modify, or replace existing pedagogical methods [12,27], and the ability of GenAI-enhanced teaching to support clinical reasoning development in areas of increased complexity and uncertainty [25]. The findings from this review will offer a pragmatic overview for all stakeholders in health care education of the existing use of GenAI-enhanced teaching methods for clinical reasoning, how these compare to traditional teaching approaches, and where the focus of future studies in this area should be targeted.

Traditional teaching methods can be limited in their ability to support the translation of clinical reasoning concepts into the clinical environment [11]. GenAI offers much promise in supporting the enhancement of clinical reasoning across a range of pedagogical approaches in health care education [11,13,17]. The evolving ability of LLMs can be harnessed to create virtual patient simulations that can be curated for complexity [35,40], support interactive learning during problem- and case-based learning [20,41] and offer formative feedback that is at least equitable to that of experts [23].

However, this exciting potential is tempered by persistent concerns about the accuracy of GenAI, its potential to stifle cognitive processes [34], foster overreliance on GenAI-produced outputs [25], or support sustainable transfer of learning into clinical practice [38]. Clinical reasoning, which is frequently described as a distinct entity, comprises a variety of skills, actions, clinical performance processes, and outcomes designed to support patient management [27]. This multilayered conceptualization is matched by associated heterogeneity in assessment approaches, leading to potential malalignment in pedagogical approaches [1]. Existing ambiguity around the defining elements of clinical reasoning and the content validity of assigned assessment approaches is a key consideration when evaluating how GenAI-enhanced teaching approaches should be used and how their success or otherwise should be interpreted.

Clear reporting structures across the domains of clinical reasoning and GenAI integration are required within studies to prevent misinterpretation or potential conflation of effect and to support the most effective integration of GenAI into existing and future teaching methods [1,12]. The findings from this study will support a rational approach to integrating GenAI for supporting clinical reasoning development by using the framework suggested by Gordon et al [1]. This framework will identify whether clinical reasoning, or components thereof, have been defined, whether teaching approaches are underpinned by an applied theory or learning objective, and the choice of assessment approach and its associated content validity [1]. Evaluating and synthesizing the existing evidence using this transparent framework will support educators and education researchers in aligning the integration of GenAI with their own educational contexts and with their own theoretical paradigms.

The integration of GenAI into health care education has accelerated at pace, with research in this field rapidly proliferating in tandem with the ongoing sophistication and fidelity of GenAI tools [42]. A scoping review by Gordon et al [12] highlighted consistent gaps in how studies were reporting the integration of AI into medical education, with isolated and fragmented information limiting replicability for future studies. A structured framework was therefore suggested as a way of systematically reporting the key elements of AI integration in future studies. This framework was abbreviated as FACETS and includes the form of AI used, the AI use case, the context of application, the education focus, a clear description of the technology and how the SAMR framework has been interpreted [58].

Although the FACETS framework was primarily devised for medical education, it is applicable across all health care education contexts. The findings from this review will offer a firmer understanding of how GenAI integration is being applied across different educational contexts and where gaps in reporting continue to exist.

### Limitations

There are several limitations that should be considered in this protocol. Determining what constitutes clinical reasoning is complex and open to risk of confirmation bias. We have attempted to mitigate this by adopting a broad definition of clinical reasoning from a widely cited study [2] and by ensuring independent review of all papers at title, abstract, and full-text screening stages, with arbitration by a third reviewer from a different clinical background. It is recognized, however, that individual perceptions will differ depending on professional background and the results of the full review should be interpreted in that light.

After completing an initial trial search including specific AI platforms, which yielded a high proportion of studies that did not meet the study eligibility criteria, the final search strategy was simplified to widen the search for relevant papers. It is recognized that the use of more generic terms in the search strategy may have missed papers looking more specifically at named AI platforms. However, given the ongoing evolution within this research space, it is anticipated that the inclusion of broader terms related to AI and GenAI within the title and abstract search strategy should widely capture the relevant evidence base, alongside an established alert put in place on Google Scholar.

### Conclusions

GenAI is already being embedded into health care education, despite consistent recognition of the limitations highlighted in existing studies [38] and despite sparse evidence of its impact on fundamental concepts such as clinical reasoning [42]. This review aims to provide a clear synthesis, using the FACETS framework [12] and a defined clinical reasoning framework [1], to inform the integration of GenAI into current and future health care curricula to support clinical reasoning development.

## Appendix

Multimedia Appendix 1 PRISMA-P checklist.

Multimedia Appendix 2 PRISMA-S checklist.

Multimedia Appendix 3 Initial search strategy.

Multimedia Appendix 4 Final search strategy.

## Abbreviations

FACETS: form of GenAI, AI use case, context, education focus, technology, SAMR
GenAI: generative AI
GRADE: Grading of Recommendations Assessment, Development, and Evaluation
LLM: large language model
PICO: population, intervention, comparator, and outcome
PRISMA: Preferred Reporting Items for Systematic Reviews and Meta-Analyses
PRISMA-P: Preferred Reporting Items for Systematic Reviews and Meta-Analysis Protocols
PRISMA-S: Preferred Reporting Items for Systematic Reviews and Meta-Analyses literature search extension
RCT: randomized controlled trial
ROB 2: Risk of Bias 2
ROBINS-I: Risk of Bias in Non-Randomized Studies of Interventions
SAMR: substitution, augmentation, modification, and redefinition
SMD: standardized mean difference
