# Supplementary material for: Effectiveness of Generative AI–Enabled Teaching for Clinical Reasoning in Health Care Education: Protocol for a Systematic Review and Meta-Analysis
Source: JMIR Res Protoc. 2026 Oct 15;15:e95709. doi: 10.2196/95709 (PMC13720408; doi:10.2196/95709)
Supplement: Supplementary file 3 [file resprot_v15i1e95709_app3.docx]

Ovid MEDLINE(R) ALL <1946 to December 16, 2025>

1	Generative Artificial Intelligence/	1348

2	((artificial intelligence or AI) adj3 (chat* or generat*)).mp. [mp=title, book title, abstract, original title, name of substance word, subject heading word, floating sub-heading word, keyword heading word, organism supplementary concept word, protocol supplementary concept word, rare disease supplementary concept word, unique identifier, synonyms, population supplementary concept word, anatomy supplementary concept word]	9071

3	((large or natural or general or deep learning) adj3 (language or text) adj3 model*).mp. [mp=title, book title, abstract, original title, name of substance word, subject heading word, floating sub-heading word, keyword heading word, organism supplementary concept word, protocol supplementary concept word, rare disease supplementary concept word, unique identifier, synonyms, population supplementary concept word, anatomy supplementary concept word]	11382

4	(GenAI or AlexaTM or (Amazon* and Alexa) or Anthropic or Bard or Bardeen or BERT or "Bing chat" or BioGPT or BLOOM or BloombergGPT or Cerebras-GPT or ChatGPT* or "Chat GPT" or chatbot* or Chatsonic or Chinchilla or Claude or DALL-E or EinsteinGPT or Ernie or Falcon or Galactica or "Generative Fill" or "GitHub Copilot" or GLaM or "Google* Assistant" or "Google* Bard" or "Google* Gemini" or Gopher or GPT-1 or GPT-2 or GPT-3* or GPT-4* or GPTNeo or GPT-NEoX or GPT-J* or "IBM Watson" or LaMDA or LLaMA or "Megatron-Turing NLG" or "Microsoft* Bing" or "Microsoft* Copilot" or Midjourney or Minerva or NeevaAI or Nvidia or OpenAI or "Open AI" or OpenAssistant or PaLM or PanGu-E or PathAI or "Path AI" or Perplexity or "pre-trained transformer*" or "pretrained transformer*" or (Apple* and Siri) or SlackGPT or "Stable Diffusion" or StyleGAN or Synthesia or XLNet or YaLM 100B or YouChat or DeepSeek or Grok or Elicit or Perplexity or "conversational agent*" or "conversational AI" or "AI assistan*" or "digital assistan*").mp. [mp=title, book title, abstract, original title, name of substance word, subject heading word, floating sub-heading word, keyword heading word, organism supplementary concept word, protocol supplementary concept word, rare disease supplementary concept word, unique identifier, synonyms, population supplementary concept word, anatomy supplementary concept word]	152155

5	1 or 2 or 3 or 4	160826

6	health personnel/ or exp allied health personnel/ or dental staff/ or exp dentists/ or exp nurses/ or nutritionists/ or occupational therapists/ or optometrists/ or pharmacists/ or physical therapists/ or exp physicians/ or exp oncologists/ or ophthalmologists/ or exp pediatricians/ or physiatrists/ or physicians, family/ or physicians, primary care/ or physicians, women/ or psychiatrists/ or pulmonologists/ or exp radiologists/ or rheumatologists/ or exp surgeons/ or urologists/	464221

7	psychologists/ or psychotherapists/	426

8	(Medic*or nurs* or dent*or pharmac* or "allied health care professional*" or AHP* or "Arts therapist*" or chiropodist* or podiatrist* or dietician* or "hearing aid dispenser*" or "occupational therapist*" or "operating department practitioner*" or orthotist* or radiographer* or "Speech language therapist*" or "speech therapist*" or orthoptist* or paramedic* or physiotherapist* or "physical therapist*" or "practitioner psychologist*" or prosthetist* or "allied health professional*" or doctor* or GP*).mp. [mp=title, book title, abstract, original title, name of substance word, subject heading word, floating sub-heading word, keyword heading word, organism supplementary concept word, protocol supplementary concept word, rare disease supplementary concept word, unique identifier, synonyms, population supplementary concept word, anatomy supplementary concept word]	513109

9	6 or 7 or 8	921442

10	exp Clinical Decision-Making/	20863

11	Metacognition/	2531

12	((Clinical or analytic* or situation* or therapeutic or diagnostic) adj3 (reasoning or judgement or competenc* or decision* or "decision making" or "problem solving" or expert*)).mp. [mp=title, book title, abstract, original title, name of substance word, subject heading word, floating sub-heading word, keyword heading word, organism supplementary concept word, protocol supplementary concept word, rare disease supplementary concept word, unique identifier, synonyms, population supplementary concept word, anatomy supplementary concept word]	258745

13	(metacognit* adj3 (skill* or activit* or process*)).mp. [mp=title, book title, abstract, original title, name of substance word, subject heading word, floating sub-heading word, keyword heading word, organism supplementary concept word, protocol supplementary concept word, rare disease supplementary concept word, unique identifier, synonyms, population supplementary concept word, anatomy supplementary concept word]	1007

14	(self-regulation or "self-regulation learn*").mp. [mp=title, book title, abstract, original title, name of substance word, subject heading word, floating sub-heading word, keyword heading word, organism supplementary concept word, protocol supplementary concept word, rare disease supplementary concept word, unique identifier, synonyms, population supplementary concept word, anatomy supplementary concept word]	13493

15	(regulat* adj3 (practi* or skill* or activit* or plan* or evaluat*)).mp. [mp=title, book title, abstract, original title, name of substance word, subject heading word, floating sub-heading word, keyword heading word, organism supplementary concept word, protocol supplementary concept word, rare disease supplementary concept word, unique identifier, synonyms, population supplementary concept word, anatomy supplementary concept word]	209068

16	10 or 11 or 12 or 13 or 14 or 15	484952

17	5 and 9 and 16	1104

18	limit 17 to yr="2021 -Current"	890

19	education/ or exp education, professional/	372502

20	((Medic*or nurs* or dent*or pharmac* or "allied health care professional*" or AHP* or "Arts therapist*" or chiropodist* or podiatrist* or dietician* or "hearing aid dispenser*" or "occupational therapist*" or "operating department practitioner*" or orthotist* or radiographer* or "Speech and language therapist*" or "speech therapist*" or orthoptist* or paramedic* or physiotherapist* or "physical therapist*" or "practitioner psychologist*" or prosthetist* or "allied health professional*" or doctor* or gp*) adj3 (educat* or training or learning)).mp. [mp=title, book title, abstract, original title, name of substance word, subject heading word, floating sub-heading word, keyword heading word, organism supplementary concept word, protocol supplementary concept word, rare disease supplementary concept word, unique identifier, synonyms, population supplementary concept word, anatomy supplementary concept word]	12289

21	19 or 20	381148

22	6 and 19	46646

23	21 or 22	381148

24	5 and 16 and 23	547

25	18 and 24	128
