# Supplementary material for: Effectiveness of Generative AI–Enabled Teaching for Clinical Reasoning in Health Care Education: Protocol for a Systematic Review and Meta-Analysis
Source: JMIR Res Protoc. 2026 Oct 15;15:e95709. doi: 10.2196/95709 (PMC13720408; doi:10.2196/95709)
Supplement: Supplementary file 4 [file resprot_v15i1e95709_app4.docx]

Scopus – final search – 05/02/26

Scopus SR final 050226

(TITLE-ABS-KEY("generative artificial intelligence" or "generative ai" or "artificial intelligence" or AI or "large language model*" or LLM or "generative language model*" or ChatGPT or GPT or "conversational agent*" or "digital assistant" or chatbot*) AND TITLE-ABS-KEY(("health care professional*" or "health care professional*" or "allied health professional*" or medic* or nurs* or dentist* or pharmac*) W/3 education) AND TITLE-ABS-KEY((clinical or analytic* or situation* or therapeutic or diagnostic) W/3 (reasoning or judg?ment or competenc* or decision* or "problem solving" or expert* or educat* or learn*))) AND PUBYEAR > 2021 AND PUBYEAR < 202

Supplementary google search – 06/02/26

Search terms used

*Generative artificial intelligence AND healthcare professional OR medical OR nursing AND clinical reasoning OR education*

Total number of studies: 85,000

Search filter of 2022-2026 – 17,300

First 500 studies checked for relevance: 1-200 – copied

200-500 checked – additional 2 studies yielded

CENTRAL – final search – 05/02/26

Ovid MEDLINE(R) ALL <1946 to February 03, 2026> (05/02/26)

1	artificial intelligence/ or generative artificial intelligence/	63829

2	("generative artificial intelligence" or "generative ai" or "artificial intelligence" or AI or "large language model*" or LLM or "generative language model*" or ChatGPT or GPT or "conversational agent*" or "digital assistant" or chatbot*).mp. [mp=title, book title, abstract, original title, name of substance word, subject heading word, floating sub-heading word, keyword heading word, organism supplementary concept word, protocol supplementary concept word, rare disease supplementary concept word, unique identifier, synonyms, population supplementary concept word, anatomy supplementary concept word]	190029

3	1 or 2	190029

4	exp education, dental/ or exp education, medical/ or exp education, nursing/ or exp education, pharmacy/	319517

5	(("health care professional*" or "health care professional*" or "allied health professional*" or medic* or nurs* or dentist* or pharmac*) adj3 education).mp. [mp=title, book title, abstract, original title, name of substance word, subject heading word, floating sub-heading word, keyword heading word, organism supplementary concept word, protocol supplementary concept word, rare disease supplementary concept word, unique identifier, synonyms, population supplementary concept word, anatomy supplementary concept word]	319693

6	4 or 5	383150

7	exp Clinical Decision-Making/	21160

8	((clinical or analytic* or situation* or therapeutic or diagnostic) adj3 (reasoning or judg?ment or competenc* or decision* or "problem solving" or expert* or educat* or learn*)).mp. [mp=title, book title, abstract, original title, name of substance word, subject heading word, floating sub-heading word, keyword heading word, organism supplementary concept word, protocol supplementary concept word, rare disease supplementary concept word, unique identifier, synonyms, population supplementary concept word, anatomy supplementary concept word]	299757

9	7 or 8	302584

10	3 and 6 and 9	1203

11	limit 10 to yr="2022 -Current"	1052

CINAHL – final search 05.02.26

Top of Form

Expanded search string

(MH "Decision Making, Clinical+" OR ((clinical or analytic* or situation* or therapeutic or diagnostic) N3 (reasoning or judg?ment or competenc* or decision* or "problem solving" or expert* or educat* or learn*))) AND (MH "Education, Medical+" OR MH "Education, Allied Health+" OR MH "Education, Nursing+" OR MH "Education, Dental" OR MH "Education, Pharmacy" OR (("health care professional*" or "health care professional*" or "allied health professional*" or medic* or nurs* or dentist* or pharmac*) N3 education)) AND (MH "Artificial Intelligence, Generative+" OR ("generative artificial intelligence" or "generative ai" or "artificial intelligence" or AI or "large language model*" or LLM or "generative language model*" or ChatGPT or GPT or "conversational agent*" or "digital assistant" or chatbot*))

Proximity;Apply equivalent subjects

Databases

CINAHL Ultimate

Bottom of Form
